# Aesthetic experience models human learning

**DOI:** 10.3389/fnhum.2023.1146083

**Published:** 2023-05-02

**Authors:** G. Gabrielle Starr

**Affiliations:** Department of Neuroscience and English, Pomona College, Claremont, CA, United States

**Keywords:** neuroaesthetics, learning, aesthetics, pleasure, preference, default mode network, salience network, central executive network

## Abstract

Aesthetic experiences have the potential to promote learning and creativity by enhancing the ability to understand complexity and to integrate novel or disparate information. Offering a theoretical framework for understanding the cognitive benefits of aesthetic experiences, this paper argues they are the necessary outcome of human learning, in which natural objects or artworks are evaluated in a multi-dimensional preference space shaped by Bayesian prediction. In addition, it contends that the brain-states underlying aesthetic experiences harness configurations of the apex three transmodal neural systems—the default mode network, the central executive network, and the salience network—that may offer information-processing advantages by recruiting the brain’s high-power communication hubs, thus enhancing potential for learning gain.

## 1. Introduction

Aesthetic experiences are nearly universal in human life—across time, age, and culture; these affective, emotional, and cognitive engagements emerge in response to nature (landscapes and faces) as well as to the arts (music, drama, literature, painting, film, dance, etc.) (Chatterjee, 2014). They are hedonic in nature—necessarily positive or negative—and require a judgment, whether conscious or not, about how an object feels to a subject, often described as how “moving” the object seems (Hume, 1999; Prinz, 2004; Menninghaus et al., 2015). Aesthetic experiences also integrate information that spans the senses and can include responses to purely imagined circumstances or even abstract ideas, as with fiction. Their universality suggests that they bring broad and significant benefits, including mood regulation (Moore, 1981; McCraty et al., 1998; Rudd et al., 2012; Zhang et al., 2014; Koelsch and Jäncke, 2015), increased empathetic abilities (Keen, 2007; Mar and Oakley, 2008; Kidd and Castano, 2013), alleviation of caregiver stress (Scrima et al., 2022), lowered blood pressure and stress (Mastandrea et al., 2019); improved health and healing (Ulrich, 1984; Ulrich et al., 2008); stronger concentration (Haake, 2011), and even increased creativity (Welke et al., 2021). Some of these benefits may accrue from enhanced positive affect (Ulrich, 1979). However, positive affect alone is not sufficient to account for all of the benefits proposed to accompany aesthetic experiences, especially those involving cognitive effects, like enhanced concentration or creative production. Finding further evidence to support these purported benefits can be well served by a focused theoretical framework. This essay offers such a framework in both a primarily psychological/computational account—aesthetic experience emerges through processes of learning based on Bayesian prediction—and a neural account—aesthetic experience emerges by way of a subset of configurations of brain systems that enable computational efficiencies and may enhance opportunities for learning.

Humans are designed to explore a dynamic world, and to do so, need to be open to multiple salient incentive possibilities from moment to moment. This means humans frequently make decisions in reference to a dynamic set of situationally-salient incentives: these form a multi-dimensional preference space which is learned—assembled over time and by experience (Section “2. Variation in aesthetic pleasure is an outcome of a multidimensional preference space”). Aesthetic preferences are learned, and they are spontaneously assembled through processes of Bayesian prediction (estimations of probability based on past experience) about both the form of objects and the sequence of events (Section “3. Aesthetic experience is an emergent property”). Aesthetic experience then emerges as a necessary outcome of the way humans learn and the parameters of human learning. Bayesian prediction and predictive processing (neural computations that estimate prediction error and anticipate likely outcomes) provide a basis for a unifying explanation of a wide range of aesthetic phenomena (Section “4. Aesthetics, expectation, and Bayesian prediction”), and can help explain why aesthetic experience supports exploratory learning (Section “5. Aesthetic pleasures are often novel and exploratory”) The pleasure signal that emerges with aesthetic experience can, especially when it is intense, precede (perhaps even triggering) changes to the configuration of the brain’s apex transmodal networks—the default mode network, the central executive network, and the salience network—that may offer computational advantages (Section “6. Aesthetics and apex transmodal neural systems”). Across these intense aesthetic experiences, much as in episodes of divergent creativity, the apex transmodal networks enable the balancing of salient externally-derived information with internal cues. Through these underlying brain states, aesthetic experience may enhance individuals’ ability to represent complexity and find novel connections among ideas, thus enhancing the possibilities for learning even as they model the way learning occurs (Section “7. Aesthetic experiences and creativity share potential for hybrid focus and similar brain states”).

## 2. Variation in aesthetic pleasure is an outcome of a multidimensional preference space

One of the most important clues to the role aesthetic experience plays in human cognition comes in the ways people differ in their aesthetic judgments. Individuals find pleasure across multiple dimensions in aesthetic experiences, especially in response to works of art (Chatterjee, 2014). For example, in listening to a piece of music, the tempo can be pleasing, as can elements of harmony or melody. The particular skill of a musician might also appeal. In addition to elements of artistic form like these, components of an individual’s own responses, like emotions or thoughts, may be pleasurable. One might savor sadness in response to a piece of music, or enjoy visualizing elements of a novel or poem (Frijda and Sundararajan, 2007; Belfi et al., 2018b). Such a range of vectors, feeding into preference evaluation, creates a multi-dimensional preference space, and that dimensionality leads to significant divergence in taste across individuals. For example, for visual art, one study showed that some individuals responded most positively to artworks that evoked feelings of awe, while others responded preferentially to those works that engendered fear, and brain signals in core arousal and emotional processes (e.g., in the pontine reticular formation) may contribute to these variations (Rudd et al., 2012; Vessel et al., 2012; Takano and Nomura, 2022). In the auditory domain, distinct behavioral and neural patterns differentiate individual taste driven by instrument type, as opposed to musical tempo (Kornysheva et al., 2010).

While there are differences in *what* about objects and artworks influences individual taste, there are also differences in *how* individuals make evaluations of these objects and artworks, and much of this difference emerges from experience. For example, human faces tend to generate high levels of agreement about attractiveness (Aharon et al., 2001). However, a study on identical twins shows that the deviation from such average agreement is more attributable to environment than to genetics (Germine et al., 2015). Accordingly, disagreement among individuals is higher for artifacts of culture than for natural objects (Vessel et al., 2018). Variance partitioning further clarifies this divergence in taste: repeated variance across individuals (variance which is not statistical noise) can be divided into the proportion that is shared and the proportion that is individual: for faces, shared variance accounts for 66% of the whole; for natural landscape scenes, 29%. For interior architecture it drops to 12%; exterior architecture, 11%; and for visual artworks like paintings, 8% (Vessel et al., 2018). The relatively high agreement around faces suggests that shared information is being used to evaluate them—most probably information that is coded closely by evolutionary considerations (e.g., those described by Rhodes, 2006). The disagreement around artifacts of culture suggests divergent sources of information; this knowledge is learned over time as individuals accrue information about styles of painting or, in a different example, the style or subject matter of poems as generically distinct as sonnets (in English, 16 lines, with a variety of typical patterns of rhyme and rhythm and a range of subjects) or haiku (in English, these generally unrhymed short poems focus on natural scenes) (Belfi et al., 2018b).

The array of features that may contribute to an aesthetic judgment is quite large. Some of these are objective features, having to do with an artwork itself. For example, Iigaya et al. (2021) identify 13 visual features of abstract and impressionist art. For poetry, such objective characteristics obviously will differ, including, for example, assonance, pitch contours, and meter (Sharinger et al., 2022). There are also subjective factors that contribute to aesthetic evaluations, including elements that are not specifically aesthetic, like everyday emotions, as well as elements more particular to aesthetic life, like melodiousness, harmony, the vivacity or vividity of mental imagery, or emotions with a particularly aesthetic cast (Zentner et al., 2008; Vessel et al., 2012; Menninghaus et al., 2015; Starr and Belfi, 2020). In addition, social elements of aesthetic engagement can bring pleasure, as can the capacity for action. For example, synchronization to a beat with music can yield the pleasure of shared movement and dancing. Music listening may also be motivated by the desire to alter one’s mood, while viewers frequently turn to visual art to learn about art and culture (Miu et al., 2016).

Factor analyses vary in indicating the number of components needed to account for variation across individuals in aesthetic judgments, with some touching on the double digits (Zentner et al., 2008; Silva and Barona, 2009). Mathematical modeling can demonstrate how integration of such disparate vectors of aesthetic pleasure or displeasure is possible. Iigaya et al. (2021) use a linear summation that integrates 13 features to generate a predictive model of the preferences individual viewers, on average, show for a set of impressionist and abstract artworks. They identified 9 low-level visual features (e.g., brightness contrast, width/height ratio, blurring, and the presence of a human figure), as well as 4 higher-order features (abstract/concrete, dynamic/still, hot/cold, and positive/negative emotion). Their model works reasonably well, predicting average preference ratings at 0.46 or 0.44 (with 0.0 being chance performance and 1.0 perfect prediction). However, the average ratings given by all observers are better at predicting preference than their model; this suggests the importance of other factors and/or processes in taste. In other words, even though they cannot identify all of the elements driving preference, Iigaya et al. (2021) convincingly show that the human brain generates aesthetic preferences at least in part by summing over several component features, and it is thus possible to begin to understand how aesthetic judgments might integrate over a multidimensional preference space. Such a preference space has the potential for numerous, divergent outcomes, depending on an individual’s weighting of each factor.

## 3. Aesthetic experience is an emergent property

Using a deep convolutional neural network (DCNN) as a model, Iigaya et al. (2021) demonstrate that higher order features can emerge by linear summation of lower order ones. In other words, it is possible that some elements of aesthetic experience—aesthetic judgments and the pleasures that go along with them—are spontaneously generated by Bayesian processes of inference. A neural network like the one used by Iigaya et al. (2021) models cognition based on the Bayesian hypothesis: the idea that a probabilistic framework is the best way to speed up information processing under conditions of uncertainty (Friston, 2010; Simonyan and Zisserman, 2015; Jospin et al., 2022). A Bayesian framework gives a clean solution to a fundamental problem: all perceptual experience is indirect, built from inferences about the relationship between sense data and the “true” state of the surrounding world. Much of that data is ambiguous, and a lot depends on an organism’s ability to resolve that ambiguity correctly. Without *a priori* knowledge, all of the conclusions an organism draws are probabilistic. Researchers increasingly believe that these inferences are best explained by positing that human cognition functions from the perspective of a Bayesian observer, using a normal distribution to determine whether or not a particular interpretation of the data (a “belief” about the world) is accurate, based on how frequently one has encountered a similar circumstance (Knill and Pouget, 2004; Griffith et al., 2008).

A number of recent accounts have suggested that Bayesian learning is central to aesthetic phenomena, and two key classes of Bayesian prediction are central: predictions about events in sequence, and predictions about perceptions (Van de Cruys and Wagmans, 2011; Cheung et al., 2019; Sarasso et al., 2020; Brielmann and Dayan, 2022; Van de Cruys et al., 2022). From an information processing perspective, an important question about perception is how an observer can reliably interpret sense data as signal, rather than noise. Quite early in the cognitive neuroscience of aesthetics, researchers argued that aesthetic pleasure attended (as the result of or coincident with) successful identification of a percept. This could take the form of matching a percept to a prototype, or, as Ramachandran and Hirstein (1999) argue, as part of a feedback loop surrounding learning. They contend that certain preferences spontaneously emerge because of the high cost of signal loss. For example, they argue the emergence of caricature in art was the result of a “peak-shift” phenomenon, whereby exaggerated features enable an observer to discriminate between competing possibilities, thus reliably identifying something as signal, rather than noise. A classic result of a peak-shift phenomenon is the sweep of a swallow’s tail, a distinctive characteristic which is hard to mistake, enabling other swallows to reliably identify a candidate for mating, rather than their risking futile effort in pursuit of a similarly sized or colored bird. Over time, processes of natural selection favor an increasingly exaggerated version of the distinguishing feature, so that if one were to graph the length of swallowtails in each generation, the “peak” in the distribution would continually shift toward the extreme. Ramachandran and Hirstein (1999) suggest a similar process at play with artistic style, as with the distortions of human figures encountered in cubism, or the proportions of female figures in classical Indian art.

While this does not give a fully credible account of the complexities of artistic style (e.g., style involves far more than exaggerated, “stylized” formal elements), the application of signal theory to aesthetic judgments suggests, importantly, that the demands of learning have implications for what individuals find aesthetically pleasing and why. While Ramachandran and Hirstein (1999) do not suggest a mechanism whereby this phenomenon might result in aesthetic pleasure, Bayesian inference can help. Indeed, the spontaneous emergence of visual preference features in Iigaya et al.’s (2021) DCNN can serve as evidence that aesthetic experiences are a necessary outcome of the way human learning works, not just of how weighted values might come together. This is because of the ways Bayesian inferences work alongside pleasure.

While successful prediction is generally pleasurable, not every successful sensory prediction yields conscious pleasure. The most fundamental indication that a sensory prediction is successful is that the sense data that matches it is experienced as vivid perception, rather than as hazy and indeterminate (Richards et al., 1996). Indeed, this is one way that specialists in computer vision define preference: the most probable configuration of facts that can be matched to visual data (Levesque, 1986). This principle helps make sense of the general human preference for color images—vivid color is one way humans register a perception as veridical and thus actionable (Sparkman and Austin, 1980). As Scottish philosopher David Hume (1739-40/2007) argued, calling a perception vivid is another way of saying it is believable (rather than being a dream or hallucination).

So, while vivid perception is an indicator of successful prediction, obviously not all vivid perception yields aesthetic pleasure. In specific, aesthetic experience generally (excepting that which emerges in connection with internally generated imagery—a special case considered shortly) concerns those validated sensory predictions that contribute to conscious experience of pleasure and displeasure across multiple simultaneously occurring vectors of evaluation. Conscious pleasure in general reflects the behavioral economy of motivation: as Carver and Scheier (2019) argue, felt pleasure emerges when there is a significant confirmation of expectation or a significant reduction in an error signal. In line with this view, powerful aesthetic experiences occur when individuals have an unusually successful opportunity to make sense of the world around them. To quote novelist Tartt (2016), ‘If a painting really works down in your heart and changes the way you see, and think, and feel, you don’t think, “oh, I love this picture because it’s universal.” “I love this painting because it speaks to all mankind.” That’s not the reason anyone loves a piece of art. It’s a secret whisper…. An individual heart-shock… [A] really great painting is fluid enough to work its way into the mind and heart through all kinds of different angles, in ways that are unique and very particular. “*Yours, yours, I was painted for you*.” ’ Feeling the power of art means feeling as if things suddenly make perfect sense, perhaps unexpectedly so.

## 4. Aesthetics, expectation, and Bayesian prediction

Bayesian inference has broad utility for understanding how aesthetic experience emerges and why it is pleasant. In addition to perceptual expectations about the form of objects, a second kind of Bayesian inference important to aesthetics involves expectations about sequences of events. Rather than being fully reactive to external stimuli, and waiting to “see” what happens in a given situation, sensory processes are predictive, anticipating likely outcomes to facilitate appropriate action (Clark, 2015). This is foundational to learning: indeed, learning is underpinned by the ability to both predict likely outcomes of events and behavior, and to integrate experience into those predictions. This set of predictions comes down to learned expectations. Many researchers have contended that expectation plays a crucial role in aesthetic pleasures (Huron, 2008; Van de Cruys and Wagmans, 2011; Tsur, 2012). This theory is widely seen as foundational in regard to music, where knowledge about a work or genre (like Western classical music, for example) may generate pleasurable tension that is resolved when a sequence of notes or chords reaches its proper resolution (Huron, 2008). Similar theories exist for poetry, where expectations about rhyme, meter, syntax or theme may enable a reader to predict and appreciate closure (Smith, 1968).

Theories of Bayesian learning and predictive processing help make sense of these pleasurable aesthetic phenomena (Sarasso et al., 2020; Brielmann and Pelli, 2021; Van de Cruys et al., 2022). Across different aesthetic contexts, learned conventions (e.g., rules of genre, frequently occurring sequences, or prototypical occurrences) shape predictions about what comes next, say, in a song or story, and a match of actual to expected conditions leads to pleasure. This means that pleasure correlates with an outcome—a perception—that is predicted by past experience (Schmidhuber, 2010). However, principles of Bayesian prediction also suggests that some of the strongest aesthetic experiences emerge not just alongside *successfully met expectations*, but in those moments where learning something new, a *violation of expectation*, reduces an error signal and increases predictive power (Schmidhuber, 2010; Van de Cruys and Wagmans, 2011; Van de Cruys et al., 2022). Indeed, as is clear from the observable divergence in individual taste, there is no single aesthetic optimum—an ideal against which experiences or objects are measured—which means that aesthetic experience and the learning that underpins it are not, and cannot be, about optimization to a single learned solution. Not only is aesthetic pleasure generated within a multi-dimensional preference space, but Bayesian learning involves matching to a range: does a percept fit within the parameters indicated by past experience? This is a dynamic process, and the most probable range of possibilities changes over time, adapting to new conditions over both the long term and shorter durations, like that of an individual poem or song. Bayesian learning requires updating predictions: the most important updates come when a prediction is wrong and when the way in which it is wrong clarifies the probability distribution (Knill and Pouget, 2004).

The case of music is instructive. It is quite common to find new music unpleasant. Listeners are unable to understand the relationship between notes, and it often just sounds wrong: surprises are evidence of failed predictions, and music in general is no exception. However, sometimes unexpected musical elements are pleasurable. Cheung et al. (2019) offer a solution to this paradox by exploring chord sequences taken from contemporary popular music. The first key here is that events that are deviations from expectation can be pleasurable when they do not result in a negative outcome (Huron, 2008). The second key is that Cheung et al. (2019) found that individuals experienced high pleasure when an unexpected chord came in the context of high certainty: the potential to gain information—to learn—is significant because baseline predictability was high (listeners can update a previously confident prediction).

Predictive processing has broad implications for understanding aesthetic experience, going beyond offering purchase on phenomena like expectation violation; it can enable the unification of many aesthetic phenomena and theories that had seemed contradictory. For example, Brielmann and Dayan’s model can predict the inverted u-shaped curve which, developed by Wundt (1874) and refined by Berlyne (1970), graphs the simultaneous increase of pleasure, repetition and complexity, as well as the decrease in pleasure following the peak in which boredom, confusion or frustration sets in. In an equation describing a parametric curve, they show that both a sensory reward and its expected value combine to account for the distinctive shape of the function. In Brielmann and Dayan’s hands, Bayesian predictive processing can also provide an explanation for the theory of processing fluency developed by Reber et al. (2004), which held that characteristics of an object or a percept that enable fluent analysis, like prototypicality, symmetry, or repetition/exposure, generate pleasure.

The Brielmann and Dayan model successfully accommodates aesthetic responses based on sensory experiences, and was designed primarily to describe pleasure in response to auditory and visual experience. However, literature offers a persistent challenge for models of aesthetic experience that begin with perception or sensation. Indeed, the Brielmann and Dayan model does not (and was not designed to) account for aesthetic pleasures that are not primarily sensory in nature, including many of the pleasures of literature as well as of elements of other art forms that also speak to content, like theme, character, or plot (e.g., painting, film, or opera). Even so, a processing-fluency account holds promise here: for example, the comprehensibility of an artwork or the elegance of writing might compress information, as with the celebrated order in complexity of wit [as English critic Johnson (1781) described it, “wit… may be… considered as a kind of *discordia concors*; a combination of dissimilar images or discovery of occult resemblances in things apparently unlike”].

Significantly, one of the central pleasures of reading literature that does not seem to promote fluent processing—generating mental imagery—can be understood using a Bayesian model. Mental imagery is especially conducive to pleasure as it increases in vividity (Belfi et al., 2018b). However, even the most vivid imagery experienced in reading is less vivid than actual perception. As described above, the vividity of everyday perception is understandable as the felt signal of predictive success: sensory data that matches expectations and is available for action is experienced as carrying the gloss of reality, rather than the haziness of imagination or daydream. Nonetheless, even though it is less vivid than perception, mental imagery can influence what individuals perceive. In an important experiment, Witzel et al. (2011) asked participants to remove color from images of everyday fruits and vegetables; those with normal vision overcorrected, rendering the object slightly tinted with the opposing color (e.g., red for green, or blue for yellow), suggesting that they imagined they “saw” the original color even in a grayscale image: e.g., participants added enough purple tint to a picture of banana that if one were to superimpose a faint yellow image, the colors would cancel each other out. Witzel et al. (2011) argue this is the outcome of Bayesian computation: “Because sensory signals always contain uncertainty, combining sensory evidence with prior knowledge is a useful strategy to constrain perceptual estimates. As a result of the combination of sensory signals and prior knowledge in a Bayesian ideal observer model, the perceptual estimate of the color… shifts toward the typical color of the object.” This works because the kind of everyday visual imagery Witzel et al. (2011) describe exists to make perception achievable using minimal resources.

The way that imagery functions to facilitate perception suggests another path through which Bayesian processes can underly aesthetic pleasure. Creating vivid visual images is metabolically costly, and some people seem to have conscious experience of imagery only infrequently, if at all (Marks, 1973; Brosch, 2018; Citron et al., 2020). For these reasons, imagery may seem to pose a problem for a processing fluency account of aesthetic pleasure. However, subsuming a processing fluency account within a predictive processing one resolves the difficulty. While generating complex or elaborate images is difficult, the kind of rudimentary imagery that facilitates everyday perception ought to be as effortless as possible if it is to fulfill its purpose of speeding up action and object recognition: successful imagery is diaphanous, i.e., barely noticeable in the phenomenal world. Imagery is experienced as imagery because one shouldn’t believe it—this is the obverse of Hume’s point about belief and vividity (Hume, 2007), and the role of predictive processing in perception helps explain why imagery can be generated at all, even if it is not being used to facilitate prediction or perception: readers’ imagery emerges because it is an aid to everyday perception (Starr, 2023a). Writers then may manipulate the wispy, diaphanous nature of mental imagery to produce aesthetic pleasure by deploying techniques to enhance vividity, even as these images remain fundamentally ghostlike (Scarry, 2001). Scarry notes that writers preferentially deploy imagery of light and shadow, for example, because it is easier to generate than more solid or substantive constructs. However, in an understanding of aesthetic experience informed by Bayesian principles of predictive processing, it is clear both why it is easier to construct such diaphanous images and why their construction may be pleasurable even if the images themselves are not vivid: in everyday perception, imagery is successful (and hence has the potential to be pleasurable) because it is diaphanous, constructed in order to be barely noticeable as it is meant to be the precursor to perception itself. Imagery helps in learning about and exploring the world, and its pleasures come when it fulfills that purpose.

## 5. Aesthetic pleasures are often novel and exploratory

In general, the kind of learning aesthetic experience models involves exploration, and again the observable divergence of taste across individuals is significant for understanding why this is the case. The broadly shared nature of aesthetic judgments of natural objects like faces or landscapes implies that in arriving at them, individuals are evaluating similar information in similar ways. As value judgments, these evaluations probably encode information that has generalizable use. For example, a spring landscape of greenery and blooms suggests abundance of food and water. Particular visual components of the landscape may be used to arrive at this judgment, including not just the presence of flowers, but the brightness of the green that belongs to young leaves. Summing over the variety of visual input driving this kind of evaluation is complex, but it is far simpler than the computation that would add autobiographical, social, or specialized knowledge to the evaluation of a painting of such a landscape.

As rapidly occurring indicators of complex analyses, aesthetic pleasures can help make sense out of complexity, conveying a generalized sense of comprehensibility or order that obtains even before individuals can consciously account for it (given the speed of aesthetic judgments, as described below). Indeed, aesthetic pleasure can influence the extraction of patterns from sense data, thus helping to organize novel information. In one remarkable example, Roeske et al. (2020) demonstrated that with listening to an unfamiliar excerpt of birdsong, listeners can parse the sequence in accordance with individually, often implicitly defined, preferred tempos, rather than the objective tempo of the birdsong itself. Here, pleasure guides learning, but not because it emerges through traditional reinforcement. In the case of reinforcement, an organism is conditioned by reward and comes to value the behavior, object, or situation that was rewarded. In this case, once an individual has identified something (here a rhythm) as pleasurable, the brain scans the environment, and uses a pleasurable interpretation as a heuristic to describe what it finds. The pleasure that comes with the perception of order may be one reason that researchers have sought to connect the “a-ha” moment with evaluations of beauty, or why, for example, scientists like physicist Paul Dirac argue that “Physical laws should have mathematical beauty,” a beauty of “universality, simplicity, inevitability, and… elemental power” (Farmelo, 2002; Korovkin et al., 2021).

Given the information processing implications of aesthetic experience, it makes sense to argue, along with Van de Cruys and Wagmans (2011), that people “engage with art because of the sense-making (regularity-revealing) value it generates.” In general, Bayesian theories of aesthetics tend to focus on predictions about how the sense data/artwork itself unfolds or is shaped: e.g., what follows in a sequence of chords. However, artworks can sequence more than percepts. In the case of literature, of course, the imagined actions of characters or the events of a narrative may be subject to a conscious version of Bayesian analysis and aesthetic judgment. But predictive processing can help generate a variety of heuristics that matter for art: in the case of music, for example, aesthetic pleasure can enable the perception of order for the music itself or for non-musical actions via entrainment. Behavioral entrainment occurs when listeners extract temporal patterns and begin to move in accordance with them (Clayton et al., 2005). Take the multiple musical sequences generated across instruments in an orchestral or jazz composition: they may be unified and understood by a listener through finding their shared beat, even when individual players deviate from a baseline pattern. Dancers moving in unison without a predetermined set of steps can also synchronize with one another in this way, both anticipating each other’s moves and compensating for unexpected steps or even errors (Jones and Boltz, 1989; Clayton et al., 2005). Aesthetic pleasure derived from listening can also direct learning and organization across a group of individuals. Indeed, the use of music to coordinate shared movement from combat to hunting is ancient, and from an adaptationist perspective, quite advantageous (Kehoe, 1999; DeNora, 2000).

## 6. Aesthetics and apex transmodal neural systems

While the principles that undergird aesthetic experience share common elements with many other kinds of valuation and kinds of learning, aesthetic experiences make something of a special case. Importantly, there is a categorical difference between liking an artwork, or even disliking it, and *powerful* aesthetic experience. In the philosophical tradition, this corresponds to the idea that mere liking—preference—is different from experiencing beauty or sublimity (Kant, 1987). Physiologically, in the case of visual art, the default mode network (DMN) selectively engages in response to powerfully moving art (Vessel et al., 2012; Belfi et al., 2019). The DMN is one of three apex transmodal networks in the human brain, neural systems into which information from across sensory, reward, evaluative, and cognitive domains can converge. The DMN tends to be engaged in spontaneous thought, imagining the self and others, imagining the future and the past, and mind-wandering (Spreng et al., 2009; Andrews-Hanna et al., 2010; Moran et al., 2013). The other two apex transmodal networks are the central executive network (CEN, sometimes called the executive control network) and salience network (SN). The CEN generally directs attention for a given task, while the SN enables individuals to detect key elements of the environment as well as of one’s own emotions or body that may cue alterations in behavior.

**FIGURE 1:**
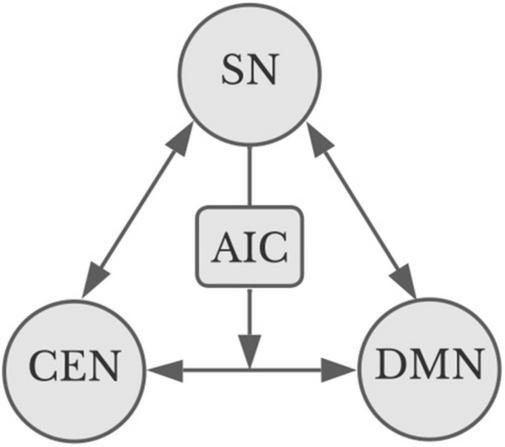
Apex transmodal networks (Starr, 2023b). The salience network (SN) can modulate and be modulated by both the central executive network and the default mode network (DMN). The CEN and the DMN interact directly: arrows indicate direction of modulation. The SN can modulate their interaction by way of the anterior insula cortex (AIC). Image credit Marilyn Perkins, adapted from Goulden et al. (2019).

As shown in Figure 1, the insula, and in particular the anterior insula cortex, plays a primary role in enabling redistribution of resources across these systems. The DMN, CEN, and SN may directly modulate one another, and the insula acts as a superhub whereby the SN may enable crosstalk between the CEN and DMN (Sridharan et al., 2008; Spreng et al., 2009, 2013; Menon and Uddin, 2010). One way to think of this is as a kind of safety protocol, enabling urgent information to rise to the top, interrupting something like reverie on the one hand (DMN) or intense concentration on a task on the other (CEN). Thus, sudden loud noises might demand one stop daydreaming and look around; equally, concentration on a delicate task can benefit from screening out distracting elements of the surrounding world.

There is evidence that crosstalk across these three apex transmodal systems is important to aesthetic experience. Cela-Conde et al. (2013) contend that aesthetic appreciation emerges via tradeoffs between the DMN, a “ventro-parietal network” and a dorsal attention network, the latter two of which map largely onto the SN and the CEN. The three networks together analyze the reward value and the emotional significance of an artwork, and the DMN engages during a time window of about 1,000−1,500 ms into visual engagement (Cela-Conde et al., 2013). Importantly, in line with this hypothesis, the insula has consistently been found to be active across aesthetic experience in different sensory modes (Brown et al., 2011). Indeed, using resting state connectivity scans, Williams et al. (2018) suggest the integration of the DMN with information from the SN is crucial in the underlying brain states across intense visual pleasure, and that differences in connectivity help drive individual differences in receptivity to engagement with art.

The particular kind of activity found with the DMN for the most powerfully pleasing visual aesthetic experience, however, has not been demonstrated with music or other art forms, even though regions in the DMN are important to aesthetic response across sensory domains. This is particularly curious given that DMN activation in intense visual aesthetic experience is primarily demonstrated in the anterior medial prefrontal cortex (amPFC), leading to the interpretation that it is involved in an assessment of the self-relevance of the artwork to the viewing individual (Vessel et al., 2012, 2013). In a separate set of experiments, researchers have confirmed that intensely appealing visual art is a special case of the self-relevance effect, whereby individuals experience enhanced memory for objects that have personal significance (Leshikar et al., 2015; Lee et al., 2023). On the other hand, Kasdan et al. (2020) have not found a similar self-relevance effect for music, which is surprising given the strong connection of music to autobiographical memory (Belfi et al., 2016).

The puzzle suggested by the absence of both DMN activation and the self-relevance effect for music makes sense, however, considering the overall demands of music experience on neural activity and the general functions of the three apex transmodal networks. The DMN was discovered as researchers sought to come to grips with some of the complexities of functional imaging. In order to understand the effects of experimental tasks on brain activity, it is necessary to understand what is happening before and after them, and resting state scans began to be essential parts of documenting functional connectivity. Because imaging methods like fMRI use metabolic processes as indirect indicators of brain function, understanding the baseline is crucial: brain metabolism is overall fairly constant, so increased activity in one region requires decreased activity elsewhere. The DMN was one of the key baseline states researchers first identified (Raichle et al., 2001; Raichle, 2015). As part of the brain’s metabolic constancy, DMN activity is generally inhibited during many demanding experimental tasks, as resources are taken for other functions.

When it comes to music, this is certainly true, offering one reason that the rebound of the DMN observed in powerful visual aesthetic experience may not regularly occur in this domain. Regions of the DMN are recruited routinely for musical analysis (Alluri et al., 2012), and such task focus can inhibit networked interactions. On the other hand, listening to familiar music *can* recruit the DMN, most probably because with a familiar tune there is lower cognitive demand in musical analysis, freeing a listener to pursue other pathways of enjoyment, including reverie or rumination (Wilkins et al., 2014; Gordon et al., 2018). In addition to the demands of sensory analysis, action preparation may also depress DMN activity, as motor planning (e.g., for production of music, dancing, or moving to the beat) is frequently an important component of listening to or imagining music (Lima et al., 2016). In all these ways, both the aesthetic object and the goals one takes to engaging it shape the ways the three apex transmodal systems interact (Starr, 2023b).

The engagement of the DMN in some—but not all—powerful aesthetic responses may also reflect the ability to prioritize pleasure in the absence of incoming salient information that requires action. Looking at a painting or leisurely listening to familiar music are generally low-risk endeavors, offering the opportunity to de-emphasize situational awareness or motor planning. The idea that aesthetic experience involves the suspension of such everyday concerns has been traditionally understood philosophically in line with the idea of an “aesthetic attitude” (Stolnitz, 1978). For example, British philosopher Burke (2015) contended in the 1750’s that individuals could only experience the most powerful of aesthetic sensations, the sublime, from a position of physical safety, because if one is personally threatened, the kinds of scenes that induce sublimity, like natural disasters, conflict, war, or even death, become too terrifying. Kant (1987) argued that powerful aesthetic responses required “disinterested” contemplation, an attitude of neutral observation, free from selfish appetites or demands. However, it is not necessary to define an attitude unique to one’s preparation for aesthetic experience. Indeed, aesthetic experiences emerge naturally from everyday neural processing (Nadal and Chatterjee, 2019). Rather, I suggest that the power of aesthetic experiences and their potential cognitive benefits emerge because the brain states underlying them provide for the swift integration of salient information across dimensions that are routinely unavailable to simultaneous or proximal access.

Sarasso et al. (2020) argue that aesthetic experience supports learning because it leads to motor inhibition, prompting an individual to stop and engage with an object, person, or experience. Processing resources are thus shifted to enable focus on the aesthetically appealing object or event. This neatly accounts for the wide range of evidence that the supplementary motor area (SMA) is activated in positive experiences with music or visual art, as well as evidence of motor potentiation with negative aesthetic experiences (Kawabata and Zeki, 2004; Di Dio et al., 2007; Brattico et al., 2013). Their account also squares with the philosophical tradition whereby powerful aesthetic experience changes the flow of daily experience, leading to an attitude of care (Heidegger, 1962; Scarry, 1999) or contemplation (Plato, 1995; Shaftesbury, 1999)—a “stopping for knowledge,” to use Sarasso et al.’s (2020) terms. However, while it may take time to absorb and assess new information, not all of the knowledge humans need to obtain requires motor inhibition alone. What it needs, rather, is motor control, which may come in the form of timing. Indeed, aesthetic pleasure can facilitate the extraction of tempo and the parsing of information, and as described above, the kind of motor coordination needed for some kinds of difficult individual endeavors or large-scale group activity can be made easier with musical accompaniment.

Moreover, understanding aesthetic responses as involving the modulation of the relative dominance of the CEN, SN, and DMN provides for a parsimonious explanation of a broad array of aesthetic phenomena, including those in which motor activity (not primarily motor inhibition) is important. The preSMA is able to act in an inhibitory fashion as part of the SN, which holds an important causal role in effecting response inhibition and cognitive control, so that stronger functional connectivity within the dorsal SN, including the preSMA, enables greater inhibitory control (Li et al., 2019). The SN enables the integration of affective and reward information to direct attention, and thus can be fundamental in knowledge acquisition.

Crucially, the brain states underlying aesthetic experience may also have computational benefits. The human brain is organized as a connectome, whereby a set of regions act like superhubs, connecting large populations of neurons (Sporns et al., 2005; Van den Heuvel and Sporns, 2011). This connectivity provides economies in signal transmission, by allowing the brain to use a high-power fast track for important signals. The recruitment of the DMN in some powerful aesthetic experience may involve a computational advantage because the default mode network uses the largest number of superhubs of any of the apex networks (Horn et al., 2014), but the absence of DMN activation in most music listening does not mean that these experiences are necessarily less emotionally or computationally powerful than with visual art. Indeed, being able to rapidly switch between the DMN, the CEN and SN in any aesthetic experience means that the brain has access to a rich range of information which can be integrated over a short period of time. There is indirect evidence for the computational potential of these states in research by Brielmann and Pelli (2021), which, using a 2-back task, demonstrated that the subjective experience of beauty could be disrupted or “knocked out” by tasks that put a significant load on working memory. In other words, if positive experiences can be foreclosed by taxing working memory, it may be because tasks that load heavily on working memory draw upon computational resources that are otherwise occupied when individuals are experiencing beauty. This is not to propose that it is working memory itself that is required for strong aesthetic experience, but rather that the overall computational intensity of such experience is high enough that these resources are not easily repurposed.

The computational efficiency of aesthetic responses is also suggested by their remarkable speed. While individuals may change their preferences over time, or might change their mind about a particular work of art, judgments of aesthetic pleasure can emerge very quickly, and there is strong evidence that these judgments can emerge even without an individual’s own conscious awareness of them, perhaps as part of an “always on” system of valuation (Nodine et al., 2008; Chatterjee et al., 2009; Lebreton et al., 2009; Mastandrea et al., 2011; Pavlović and Marković, 2012; Bohrn et al., 2014; Mastandrea and Maricchiolo, 2014). The dominant cognitivist models of aesthetics reflect this speed in the initial stages of an aesthetic encounter (Brattico, 2009; Leder and Nadal, 2014). Using continuous behavioral measures, peak pleasure in looking at visual art appears within 6 s of image onset, even for viewing times of 1, 5, or 15 s (Belfi et al., 2019). These behavioral responses emerge faster than underlying reward activity in the brain, which can peak 10 or more seconds into a visual encounter (Belfi et al., 2019). For music, individuals can arrive at a stable aesthetic evaluation in milliseconds (Brattico et al., 2013; Belfi et al., 2018a). There is also evidence from MEG, cited above, that pleasure emerges prior to DMN engagement, with a beauty judgment in milliseconds after individuals begin to view a work of visual art and DMN engagement following, at 1,000−1,500 ms (Cela-Conde et al., 2013).

The speed of aesthetic judgments offers a decided advantage for decision making under uncertainty—the primary function of Bayesian prediction—in which an individual would not have a well-developed *a priori* expectation about the outcome, as with a novel object or a novel class of objects. This in fact the case for everyday life, in which individuals have to “try it” to find out whether they like it: for this reason the commercial development of predictive tools that can be used to project the likelihood of an individual’s enjoyment of music, books, or films is so valuable, as is the case with products like the music-listening service Pandora, which in 2021 generated $2 billion USD in revenue (Curry, 2023).

**FIGURE 2:**
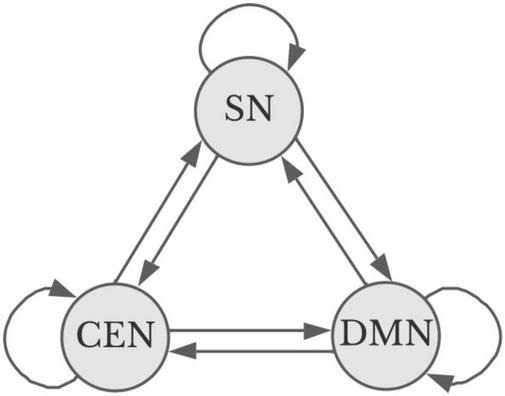
Simplified Markov diagram for apex transmodal networks (Starr, 2023b). Straight lines indicate a potential change of state favoring one or more networks. Curved lines represent the potential to remain in a given state. Sensory analysis and the particular goals motivating aesthetic encounters influence the probability of and direction for a shift in state. Image credit Marilyn Perkins.

At a macro level, given the homeostatic balance that underpins the structural relationship between the three apex transmodal networks, it should be possible to understand and predict the brain states associated with aesthetic experience using a Markov chain. Markov chains represent the probability that a system will remain in one state or move to another based on a (experimentally determined) coefficient that multiplies the baseline probabilities of change (see Figure 2). For powerful visual aesthetic experience, based on experimentation (Belfi et al., 2019), one would predict the probability that the DMN and SN will be dominant is near 1.0 within 10 s of engagement; however, with viewing paintings that individuals do not find particularly appealing, the probability early in the visual encounter of that state is near zero. Significantly, experimentation has also shown that individuals eventually revert to DMN engagement with non-preferred artworks, as they presumably disengage from the artwork and return to mind-wandering and other baseline cognitive activity (Belfi et al., 2019).

**FIGURE 3:**
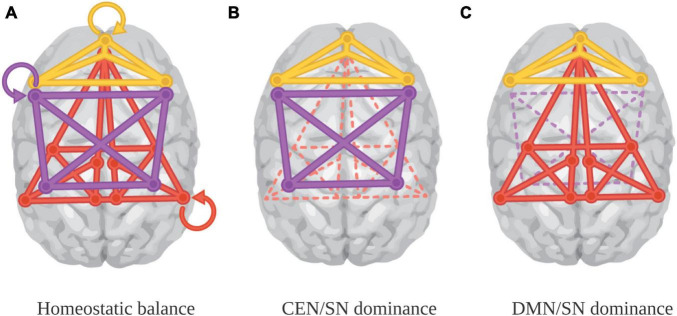
Baseline and paired-system dominance (Starr, 2023b). Panel **(A)** represents baseline connectivity. Panel **(B)** represents deactivation of the DMN in aesthetic experiences that require intensive task focus, like musical performance. Panel **(C)** represent DMN engagement in aesthetic experiences were task focus or intensive sensory analysis are not required, and self-reflection is potentiated. Image credit Marilyn Perkins. Panel **(A)** adapted from Manoliu et al. (2014).

The probability of engaging one apex system or another depends on the cognitive demands associated with a particular aesthetic experience (see Figure 3). With intense visual aesthetic experience, the rise of the DMN can enable tapping information about a range of self-relevant areas (like memories), as well as the integration of the kind of visceral knowledge usually associated with the SN (panel C). With music, however, while the DMN may not be regularly engaged outside of familiar works, what is typically integrated can include interoceptive data, including emotion, reward information, and gut feelings (for example, those associated with peak aesthetic experiences like goosebumps and chills), alongside action preparation, musical and semantic analysis, declarative knowledge, and exteroceptive information about one’s own motion and that of others—an extensive range of kinds and complexity of information (panel B).

## 7. Aesthetic experiences and creativity share potential for hybrid focus and similar brain states

In integrating salient interoceptive information with exteroceptive awareness, aesthetic experience may involve a synergistic, hybrid kind of focus. While different artforms offer different affordances (routes for engagement), the capacity of art to provoke integration of generally divergent kinds of information is remarkable. The breadth of information, though, requires a hybrid focus—an ability to hold often divergent information in mind—to integrate it. This is perhaps most apparent with literature, because the sensory input on which engagement with literature generally depends—whether visual or aural—generally is not the object of aesthetic judgment (Starr, 2015). Aesthetic evaluations here rely most heavily on internally generated data, whether it is semantic information, interpretations, imagery, or emotion. However, when formal properties (e.g., rhyme, meter, or assonance) are the immediate object of judgment, aesthetic evaluation takes place alongside the internal evaluations that give form meaning: poetry by rule is not pretty noise. Additionally, at times, something like mind-wandering may take over in immersive reading, and may involve regions in the DMN, but in others, attention to literary elements like metaphor or allusion may place cognitive demands elsewhere (Jacobs, 2015). The variety of information is important, driving the divergence of individual taste and influencing the brain states that are engaged in aesthetic experience: “encounters with art can differ because the information that is combined to produce the experience of art varies. Many of the brain regions identified by neuroimaging studies of art appreciation play a key role in integrating information derived from the diverse transient functional networks…, including the default mode network, the salience network, the executive network, and motor and sensory networks” (Nadal and Chatterjee, 2019).

There is evidence for this view—that aesthetic experience requires dual focus—as well as for the importance of aesthetics for learning, in the relationship between creativity and aesthetic experience. Aesthetic experience is fundamentally linked to creativity from the perspective of the artist, but there is evidence that the mental states that lead to creative production are also intimately connected to those of aesthetic experiences. Welke et al. (2021) demonstrate that powerful aesthetic experience can prime creative inspiration. Individuals in their study selected visual artworks that they found intensely aesthetically pleasing. Participants were then asked to use these artworks, novel artworks, or three-word triads as prompts for writing. They were also asked to rate their own “felt inspiration.” The powerfully moving art, and neither the three-word triads nor the novel artworks, correlated with higher felt inspiration and longer compositions. They also tested participants entirely on artworks, with moving, novel, and non-moving previously viewed visual art as writing prompts. The only significant effect was from the most aesthetically appealing art: familiarity itself had no effect, and strong aesthetic experience held a special place.

The behavioral connection between intense aesthetic experience and creativity is paralleled by evidence from neuroscience. Experimentation has revealed default mode activation in creative episodes, as well as enhanced connectivity between regions in the DMN, the CEN and the insula (Beaty, 2015; Liu et al., 2015; Pinho et al., 2015; Bashwiner et al., 2016). It is the cross-talk between these three apex systems that, as with aesthetic experience, also appears crucial in some kinds of creative thinking: Vartanian (2019) contends that “the interplay between the default mode network and the executive control network can be perceived as the interplay between controlled and spontaneous thought processes in the service of novel and useful idea generation.” Vartanian et al. (2018) demonstrated that areas in the inferior frontal gyrus (IFG) modulate regions in the medial temporal gyrus (MTG) in cases of divergent thought: “conceptually, this is consistent with a model wherein the IFG prunes the ideas generated by MTG to produce responses that meet task demands” (Vartanian et al., 2018). There is further evidence that the interplay between the DMN and CEN can be understood to involve balancing disinhibition and controlled direction, because disinhibition in highly creative individuals may be in part effected by way of decreased white matter thickness in parts of this circuitry (Jung et al., 2010).

Disinhibition is important in some kinds of creative thinking so that learned associations may be placed in expanded context and new possibilities perceived. In this view, in the search for a novel solution, the ability to activate multiple related ideas, memories, or constructs before pruning them is a creative advantage. This is crucial because activation of one idea generally results in inhibition of other closely related ones, so that an individual can maintain clear, focused attention. However, it is useful at times to be able to access multiple, connected ideas and hold them in thought simultaneously, as with brainstorming new ideas, and the ability to switch between diffuse and directed focus is often described as flexible cognitive control (Benedeck and Fink, 2019). Thus, another reason powerful aesthetic experiences and divergent-thought creativity may be alike is because aesthetic experiences also involve multiple, proximal possibilities for interpretation and evaluation, a subset of which coalesce and are accompanied by pleasure.

## 8. Hybrid focus, intrinsic motivation, and the potential for enhanced learning

Aesthetic experience may offer enhanced potential for learning, because like creative thought, it can function as a key to a special kind of lock, enabling synergistic integration of information across systems that are normally in competition from a psychological or neural perspective. In powerfully pleasing visual aesthetic experiences, the tradeoffs between the three apex transmodal networks result in the ability of the DMN to be active in circumstances in which it otherwise would not. Rather than focusing primarily on the task at hand (CEN) or on scanning the environment for behaviorally salient information (SN), the default mode network can support attention focused internally while an individual also focuses externally. The hybrid focus that underpins some kinds of creative thought may be similar to—though not identical with—that of powerful visual aesthetic experience because they both rely on similar brain states.

Aesthetic experience allows individuals to quickly organize and make meaning out of many of the vast kinds of information that humans encounter as they explore their environment (Schoeller, 2015). As Biederman and Vessel (2006) argue, humans are “infovores,” and aesthetically appealing objects and experiences offer pleasures that incentivize exploration (Walton, 1990). The literature around motivation devotes significant attention to the mechanisms involved in exploratory learning, whether they rely on primary, unlearned reinforcement, on motivational cues that come from the world, or on those incentives that emerge from within (Berridge, 2004; Kringelbach and Berridge, 2017). There is an extensive philosophical tradition suggesting the latter—that aesthetic experience involves intrinsic motivation. By contrast with other pleasures, in this view what distinguishes aesthetic pleasures is that they are not exhausted as individuals consume them. They are good for their own sake, neither requiring possession nor leading to satiation (Addison, 1965; Kant, 1987; Shaftesbury, 1999). Again, however, there is no need to posit a special aesthetic drive. Intrinsically motivated, curiosity-driven exploratory behavior and aesthetic experiences may be related because they both reflect a solution to the trade offs required by exploratory learning. Van de Cruys et al. (2022) contend that “learning is costly and fallible, so being able to sensitively direct resources to where the best learning progress can be made has a considerable advantage.” Aesthetic experience is one way in which humans can feel, and thus find, the “sweet spot” for learning gain.

Some researchers posit that for humans, like other mammals, exploratory behavior is supported by a generally active “seeking” system, which relies on dopaminergic neurons and supports continual scanning of the environment for behaviorally salient information (Panksepp and Biven, 2012). This system is bottom up, rather than top down; information is filtered by and evaluated within the SN, incentivizing further exploration and engagement, and even allowing for boredom (reallocation of resources/shifts in attention) as an animal moves through its environment. As humans forage for information without an explicit goal in mind—think a leisurely walk through a forest or a stroll around a city neighborhood—the SN registers the motivational significance of the information discovered. However, rather than simply scanning the environment for salient information in an open-ended way, at times individuals are alert to internally generated rewards while they pursue other explicit goals—in these cases, they are undertaking work that is intrinsically rewarding (as opposed to work whose reward is triggered by external cues). For such intrinsically motivated tasks, the SN should interact dynamically with the CEN (Figure 3, panel B) so that bottom-up information can be fed into goal-directed behavior (Ryan and Di Domenico, 2016; Di Dimenico and Ryan, 2017). The SN helps identify internally-generated information, like affect or emotion, which can then help to regulate task focus (Seeley et al., 2007). Importantly, the usual antagonism between the DMN and the CEN generally obtains in these tasks, with DMN down-regulation apparent in a variety of studies of intrinsically motivating tasks (Lee et al., 2012; Lee and Reeve, 2013; Ulrich et al., 2014). Yet, there remains a wealth of information encoded within the DMN that is useful for self- and other-directed cognition; if, as is usual in intrinsically motivated tasks, the CEN and SN pairing leads to DMN suppression, the normal computational efficiencies that enable rapid synthesis of this information no longer obtain. In other words, aesthetic experience sits in an enviable position in the tradeoffs necessary to learning about and navigating the world, because it establishes conditions whereby an individual can finely regulate salience detection to gain access, preferentially, to forms of value with inward significance, maintain focus on cognitively significant external information, and make use of high-priority routes for signal transmission.

## 9. Conclusion

Compared to other organisms, human beings have a wide range of potential preferences generated by the complexity of our evolutionary niche and our abilities to adapt to as well as mold the environment. That is, over time, not only has the human species adapted to the vast environmental differences found in life from the poles to the equator, but we continually adapt to the changes our own technologies have created. The ability to master these different environments successfully is predicated on the capacity to explore widely, directed by sensitivity to salience and reward cues that are dynamically shifting and expansive. For example, the orbitofrontal cortex, with its generalizable ability to represent reward across multiple domains (Levy and Glimcher, 2012), offers a system that can enable individuals to value events and objects as evolutionarily distant as food, music, dollars, bitcoin and video games. Because of the ability to value so many different classes and instances of objects, the vectors of preference available to human beings are truly expansive, generating the wide range of differing tastes that have been observed specifically for objects of culture (Vessel et al., 2018). Some of the preferences humans experience are aesthetic, generated in response to sense data or imagined sensations and seemingly pleasurable for their own sake. These preferences evolve spontaneously, as an outcome of Bayesian predictions about the environment.

Aesthetic experiences are particularly valuable for organisms that thrive on knowledge, because they involve computationally advantageous integrations of interoceptive and exteroceptive information across apex transmodal neural systems. Exploring the possibilities of aesthetic experiences means exercising the ability to learn in a way fundamental to human life. As Bayesian processes of inference enable the spontaneous generation of complex forms of preference, the pleasures that emerge can aid in the discovery of patterns, and thus generate further heuristics useful for organizing information. These heuristics are not just useful for sense data, but for social information too, helping individuals learn group behaviors and adapt to changes quickly. Kant (1987) argued that aesthetic judgments occur when an object perfectly fits the human capacities of cognition; in the framework offered here, aesthetic experience emerges because of the rule-bound processes of prediction that shape human learning. Aesthetic experience models fluent conditions for learning, and exploring aesthetic experience may help individuals to learn how to learn better, how to learn more productively, and even how to learn together.

## Data availability statement

The original contributions presented in this study are included in the article, further inquiries can be directed to the corresponding author.

## Author contributions

GS conceived, wrote, and approved the submitted manuscript.

## References

[B1] Addison J. (1965). *The spectator.* Oxford: Clarendon Press.

[B2] Aharon I. Etcoff N. Ariely D. Chabris C. F. O’Connor E. Breiter H. C. (2001). Beautiful faces have variable reward value: fMRI and behavioral evidence. *Neuron* 32, 537–551. 10.1016/S0896-6273(01)00491-3 11709163

[B3] Alluri V. Toiviainen P. Jääskeläinen I. P. Glerean E. Sams M. Brattico E. (2012). Large-scale brain networks emerge from dynamic processing of musical timbre, key and rhythm. *Neuroimage* 59 3677–3689. 10.1016/j.neuroimage.2011.11.019 22116038

[B4] Andrews-Hanna J. R. Reidler J. S. Sepulcre J. Poulin R. Buckner R. L. (2010). Functional-anatomic fractionation of the brain’s default network. *Neuron* 65 550–562. 10.1016/j.neuron.2010.02.005 20188659 PMC2848443

[B5] Bashwiner D. M. Wertz C. J. Flores R. A. Jung R. E. (2016). Musical creativity “revealed” in brain structure: interplay between motor, default mode and limbic networks. *Sci. Rep.* 6 1–8. 10.1038/srep20482 26888383 PMC4757893

[B6] Beaty R. E. (2015). The neuroscience of musical improvisation. *Neurosci. Biobehav. Rev*. 51, 108–117. 10.1016/j.neubiorev.2015.01.004 25601088

[B7] Belfi A. M. Karlan B. Tranel D. (2016). Music evokes vivid autobiographical memories. *Memory* 24 979–989. 10.1080/09658211.2015.1061012 26259098

[B8] Belfi A. M. Vessel E. A. Starr G. G. (2018b). Individual ratings of vividness predict aesthetic appeal in poetry. *Psychol. Aesthe. Creat. Arts* 12:153. 10.1037/aca0000153

[B9] Belfi A. M. Kasdan A. Rowland J. Vessel E. A. Starr G. G. Poeppel D. (2018a). Rapid timing of musical aesthetic judgments. *J. Exp. Psychol. General.* 147:1531. 10.1037/xge0000474 30010370

[B10] Belfi A. M. Vessel E. A. Brielmann A. Isik A. I. Chatterjee A. Leder H. (2019). Dynamics of aesthetic experience are reflected in the default-mode network. *NeuroImage* 188 584–597. 10.1016/j.neuroimage.2018.12.017 30543845 PMC8493917

[B11] Benedeck M. Fink A. (2019). Toward a neurocognitive framework of creative cognition: the role of memory, attention, and cognitive control. *Curr. Opin. Behav. Sci.* 27 116–122. 10.1016/j.cobeha.2018.11.002

[B12] Berlyne D. E. (1970). Novelty, complexity, and hedonic value. *Percept. Psychophys.* 8 279–286. 10.3758/BF03212593

[B13] Berridge K. C. (2004). Motivation concepts in behavioral neuroscience. *Physiol. Behav.* 81 179–209. 10.1016/j.physbeh.2004.02.004 15159167

[B14] Biederman I. Vessel E. A. (2006). Perceptual pleasure and the brain: a novel theory explains why the brain craves information and seeks it through the senses. *Am. Sci.* 94 247–253. 10.1511/2006.59.247

[B15] Bohrn I. C. Altmann U. Lubrich O. Menninghaus W. Jacobs A. M. (2014). When we like what we know–a parametric fMRI analysis of beauty and familiarity. *Brain Lang.* 124 1–8. 10.1016/j.bandl.2012.10.003 23332807

[B16] Brattico E. (2009). “The neuroaesthetics of music: a research agenda coming of age,” in *Oxford handbook of music and the brain*, eds. Thaut M. H. Hodges D. A. (Oxford University Press), 364–390.

[B17] Brattico E. Bogert B. Jacobsen T. (2013). Toward a neural chronometry for the aesthetic experience of music. *Front. Psychol.* 4:206. 10.3389/fpsyg.2013.00206 23641223 PMC3640187

[B18] Brielmann A. A. Dayan P. (2022). A computational model of aesthetic value. *Psychol. Rev.* 129 1319–1337. 10.1037/rev0000337 35786988

[B19] Brielmann A. A. Pelli D. G. (2021). Intense beauty requires intense pleasure. *Front. Psychol.* 10:2420. 10.3389/fpsyg.2019.02420 31749737 PMC6848232

[B20] Brosch R. (2018). What we ‘see’ when we read: visualization and vividness in reading fictional narratives. *Cortex* 105, 35–143. 10.1016/j.cortex.2017.08.020 28916259

[B21] Brown S. Gao X. Tisdelle L. Eickhoff S. B. Liotti M. (2011). Naturalizing aesthetics: brain areas for aesthetic appraisal across sensory modalities. *Neuroimage* 58 250–258. 10.1016/j.neuroimage.2011.06.012 21699987 PMC8005853

[B22] Burke E. (2015). *A philosophical enquiry into the sublime and beautiful.* Oxford: Oxford University Press.

[B23] Carver C. S. Scheier M. F. (2019). “A self-regulatory viewpoint on human behavior,” in *Oxford handbook of human motivation*, ed. Ryan R. M. (Oxford University Press). 10.1093/oxfordhb/9780190666453.013.3

[B24] Cela-Conde C. J. García-Prieto J. Ramasco J. J. Mirasso C. R. Bajo R. Munar E. (2013). Dynamics of brain networks in the aesthetic appreciation. *Proc. Natl. Acad. Sci. U.S.A.* 110 10454–10461. 10.1073/pnas.1302855110 23754437 PMC3690613

[B25] Chatterjee A. (2014). *The aesthetic brain: how we evolved to desire beauty and enjoy art.* Oxford: Oxford University Press. 10.1093/acprof:oso/9780199811809.001.0001

[B26] Chatterjee A. Thomas A. Smith S. E. Aguirre G. K. (2009). The neural response to facial attractiveness. *Neuropsychology* 23:135. 10.1037/a0014430 19254086

[B27] Cheung V. K. Harrison P. M. Meyer L. Pearce M. T. Haynes J. D. Koelsch S. (2019). Uncertainty and surprise jointly predict musical pleasure and amygdala, hippocampus, and auditory cortex activity. *Curr. Biol.* 29 4084–4092. 10.1016/j.cub.2019.09.067 31708393

[B28] Citron F. M. Lee M. Michaelis N. (2020). Affective and psycholinguistic norms for German conceptual metaphors (COMETA). *Behav. Res. Methods* 52 1056–1072. 10.3758/s13428-019-01300-7 31919761

[B29] Clark A. (2015). *Surfing uncertainty: prediction, action, and the embodied mind.* Oxford: Oxford University Press. 10.1093/acprof:oso/9780190217013.001.0001

[B30] Clayton M. Sager R. Will U. (2005). In time with the music: the concept of entrainment and its significance for ethnomusicology. *Eur. Meet. Ethnomusicol.* 11 1–82.

[B31] Curry D. (2023). *Pandora music revenue and usage statistics. Business of Apps.* Available online at: https://www.businessofapps.com/data/pandora-statistics/ (accessed January 12, 2023)

[B32] DeNora T. (2000). *Music in everyday life.* Cambridge: Cambridge University Press. 10.1017/CBO9780511489433

[B33] Di Dio C. Macaluso E. Rizzolatti G. (2007). The golden beauty: brain response to classical and renaissance sculptures. *PLoS One* 2:e1201. 10.1371/journal.pone.0001201 PMC2065898 18030335

[B34] Di Dimenico S. I. Ryan R. M. (2017). The emerging neuroscience of intrinsic motivation: a new frontier in self-determination research. *Front. Hum. Neurosci.* 11:145. 10.3389/fnhum.2017.00145 28392765 PMC5364176

[B35] Farmelo G. (2002). *It must be beautiful: great equations of modern science.* London: Granta.

[B36] Frijda N. H. Sundararajan L. (2007). Emotion refinement: a theory inspired by Chinese poetics. *Perspect. Psychol. Sci.* 2 227–241. 10.1111/j.1745-6916.2007.00042.x 26151967

[B37] Friston K. (2010). The free-energy principle: a unified brain theory? *Nat. Rev. Neurosci.* 11 127–138. 10.1038/nrn2787 20068583

[B38] Germine L. Russell R. Bronstad P. M. Blokland G. A. Smoller J. W. Kwok H. (2015). Individual aesthetic preferences for faces are shaped mostly by environments, not genes. *Curr. Biol.* 25 2684–2689. 10.1016/j.cub.2015.08.048 26441352 PMC4629915

[B39] Gordon C. L. Cobb P. R. Balasubramaniam R. (2018). Recruitment of the motor system during music listening: an ALE meta-analysis of fMRI data. *PLoS One* 13:e0207213. 10.1371/journal.pone.0207213 30452442 PMC6242316

[B40] Goulden N. Khusnulina A. Davis N. J. Bracewell R. M. Bokde A. L. McNulty J. P. (2019). The salience network is responsible for switching between the default mode network and the central executive network: replication from DCM. *Neuroimage* 99 180–190. 10.1016/j.neuroimage.2014.05.052 24862074

[B41] Griffith T. L. Kemp C. Tenenbaum J. B. (2008). “Bayesian models of cognition,” in *The Cambridge handbook of computational psychology*, ed. Sun R. (Cambridge University Press), 59–100. 10.1017/CBO9780511816772.006

[B42] Haake A. B. (2011). Individual music listening in workplace settings: an exploratory survey of offices in the UK. *Musicae Sci.* 15 107–129. 10.1177/1029864911398065

[B43] Heidegger M. (1962). *Being and time*, trans. Macquarrie J. Robinson E. (New York, NY: Harper and Row).

[B44] Horn A. Ostwald D. Reisert M. Blankenburg F. (2014). The structural–functional connectome and the default mode network of the human brain. *Neuroimage* 102 142–151. 10.1016/j.neuroimage.2013.09.069 24099851

[B45] Hume D. (1999). “Of the standard of taste,” in *Eighteenth-century British aesthetics*, ed. Townsend D., 230–241.

[B46] Hume D. (2007). *A treatise of human nature.* Oxford: Oxford University Press.

[B47] Huron D. (2008). *Sweet anticipation: music and the psychology of expectation.* Cambridge, MA: MIT Press.

[B48] Iigaya K. Yi S. Wahle I. A. Tanwisuth K. O’Doherty J. P. (2021). Aesthetic preference for art can be predicted from a mixture of low-and high-level visual features. *Nat. Hum. Behav.* 91 1699–1705. 10.1038/s41562-021-01124-6 34017097 PMC8494016

[B49] Jacobs A. M. (2015). Towards a neurocognitive poetics model of literary reading. *Cogn. Neurosci. Nat. Lang. Use* 2015 135–159. 10.1017/CBO9781107323667.007

[B50] Johnson S. (1781). *Lives of the English poets.*

[B51] Jones M. R. Boltz M. (1989). Dynamic attending and responses to time. *Psychol. Rev.* 96:459. 10.1037/0033-295X.96.3.459 2756068

[B52] Jospin L. V. Laga H. Boussaid F. Buntine W. Bennamoun M. (2022). Hands-on bayesian neural networks—a tutorial for deep learning users. *IEEE Comput. Int. Magaz.* 17 29–48. 10.1109/MCI.2022.3155327

[B53] Jung R. E. Grazioplene R. Caprihan A. Chavez R. S. Haier R. J. (2010). White matter integrity, creativity, and psychopathology: disentangling constructs with diffusion tensor imaging. *PLoS One* 5:e9818. 10.1371/journal.pone.0009818 20339554 PMC2842439

[B54] Kant I. (1987). *Critique of judgment.*, trans. Pluhar W. (Indianapolis, IN: Hackett).

[B55] Kasdan A. Belfi A. M. Grassi M. (2020). *Investigating a self-reference effect in musical aesthetics.* Cambridge: Cambridge University Press. 10.1017/exp.2020.6

[B56] Kawabata H. Zeki S. (2004). Neural correlates of beauty. *J. Neurophys*. 91, 1699–1705. 10.1152/jn.00696.2003 15010496

[B57] Keen S. (2007). *Empathy and the novel.* Oxford: Oxford University Press. 10.1093/acprof:oso/9780195175769.001.0001

[B58] Kehoe A. B. (1999). “Blackfoot and other hunters of the North American Plains,” in *The Cambridge encyclopedia of hunters and gatherers*, eds Lee R. B. Daly R. (Cambridge University Press), 36–40.

[B59] Kidd D. C. Castano E. (2013). Reading literary fiction improves theory of mind. *Science* 342 377–380. 10.1126/science.1239918 24091705

[B60] Knill D. C. Pouget A. (2004). The bayesian brain: the role of uncertainty in neural coding and computation. *Trends Neurosci.* 27 712–719. 10.1016/j.tins.2004.10.007 15541511

[B61] Koelsch S. Jäncke L. (2015). Music and the heart. *Eur. Heart J.* 36 3043–3049. 10.1093/eurheartj/ehv430 26354957

[B62] Kornysheva K. von Cramon D. Y. Jacobsen T. Schubotz R. I. (2010). Tuning-in to the beat: aesthetic appreciation of musical rhythms correlates with a premotor activity boost. *Hum. Brain Mapp.* 31 48–64. 10.1002/hbm.20844 19585590 PMC6870655

[B63] Korovkin S. Savinova A. Padalka J. Zhelezova A. (2021). Beautiful mind: grouping of actions into mental schemes leads to a full insight Aha! experience. *J. Cogn. Psychol.* 33 620–630. 10.1080/20445911.2020.1847124

[B64] Kringelbach M. L. Berridge K. C. (2017). Neuroscience of reward, motivation, and drive. *Recent Dev. Neurosci. Res. Hum. Motivat.* 19 23–35. 10.1108/S0749-742320160000019020

[B65] Lebreton M. Jorge S. Michel V. Thirion B. Pessiglione M. (2009). An automatic valuation system in the human brain: evidence from functional neuroimaging. *Neuron* 64 431–439. 10.1016/j.neuron.2009.09.040 19914190

[B66] Leder H. Nadal M. (2014). Ten years of a model of aesthetic appreciation and aesthetic judgments: the aesthetic episode–developments and challenges in empirical aesthetics. *Br. J. Psychol.* 105 443–464. 10.1111/bjop.12084 25280118

[B67] Lee H. Jacquot A. Makowski D. Arcangeli M. Dokic J. Piolino P. (2023). The beauty and the self: a common mnemonic advantage between aesthetic judgment and self-reference. *Psychol. Conscious. Theory Res. Pract.* 2023:345. 10.1037/cns0000345

[B68] Lee W. Reeve J. (2013). Self-determined, but not non-self-determined, motivation predicts activations in the anterior insular cortex: an fMRI study of personal agency. *Soc. Cogn. Affect. Neurosci.* 8 538–545. 10.1093/scan/nss029 22451482 PMC3682437

[B69] Lee W. Reeve J. Xue Y. Xiong J. (2012). Neural differences between intrinsic reasons for doing versus extrinsic reasons for doing: an fMRI study. *Neurosci. Res.* 73 68–72. 10.1016/j.neures.2012.02.010 23565014 PMC3614004

[B70] Leshikar E. D. Dulas M. R. Duarte A. (2015). Self-referencing enhances recollection in both young and older adults. *Aging Neuropsychol. Cogn.* 22 388–412. 10.1080/13825585.2014.957150 25264018 PMC4377313

[B71] Levesque H. J. (1986). Making believers out of computers. *Artifi. Int.* 30 81–108. 10.1016/0004-3702(86)90068-8

[B72] Levy D. J. Glimcher P. W. (2012). The root of all value: a neural common currency for choice. *Curr. Opin. Neurobiol.* 22 1027–1038. 10.1016/j.conb.2012.06.001 22766486 PMC4093837

[B73] Li L. M. Violante I. R. Leech R. Hampshire A. Opitz A. McArthur D. (2019). Cognitive enhancement with salience network electrical stimulation is influenced by network structural connectivity. *Neuroimage* 185 425–433. 10.1016/j.neuroimage.2018.10.069 30385222 PMC6299257

[B74] Lima C. F. Krishnan S. Scott S. K. (2016). Roles of supplementary motor areas in auditory processing and auditory imagery. *Trends Neurosci.* 39 527–542. 10.1016/j.tins.2016.06.003 27381836 PMC5441995

[B75] Liu S. Erkkinen M. G. Healey M. L. Xu Y. Swett K. E. Chow H. M. (2015). Brain activity and connectivity during poetry composition: toward a multidimensional model of the creative process. *Hum. Brain Mapp.* 36 3351–3372. 10.1002/hbm.22849 26015271 PMC4581594

[B76] Manoliu A. Riedl V. Zherdin A. Mühlau M. Schwerthöffer D. Scherr M. (2014). Aberrant dependence of default mode/central executive network interactions on anterior insular salience network activity in schizophrenia. *Schizop. Bull.* 40 428–437. 10.1093/schbul/sbt037 23519021 PMC3932085

[B77] Mar R. A. Oakley K. (2008). The function of fiction is the abstraction and simulation of social experience. *Perspect. Psychol. Sci.* 3 173–192. 10.1111/j.1745-6924.2008.00073.x 26158934

[B78] Marks D. F. (1973). Visual imagery differences in the recall of pictures. *Br. J. Psychol.* 64 17–24. 10.1111/j.2044-8295.1973.tb01322.x 4742442

[B79] Mastandrea S. Maricchiolo F. (2014). Implicit and explicit aesthetic evaluation of design objects. *Art Percept.* 2 141–162. 10.1163/22134913-00002015

[B80] Mastandrea S. Bartoli G. Carrus G. (2011). The automatic aesthetic evaluation of different art and architectural styles. *Psychol. Aesthe. Creat. Arts* 5 126–134. 10.1037/a0021126

[B81] Mastandrea S. Maricchiolo F. Carrus G. Giovannelli I. Giuliani V. Berardi D. (2019). Visits to figurative art museums may lower blood pressure and stress. *Arts Health* 11 123–132. 10.1080/17533015.2018.1443953 31038442

[B82] McCraty R. Barrios-Choplin B. Atkinson M. Tomasino D. (1998). The effects of different types of music on mood, tension, and mental clarity. *Alternat. Ther. Health Med.* 4 75–84. 9439023

[B83] Menninghaus W. Wagner V. Hanich J. Wassiliwizky E. Kuehnast M. Jacobsen T. (2015). Towards a psychological construct of being moved. *PLoS One* 10:e0128451. 10.1371/journal.pone.0128451 26042816 PMC4456364

[B84] Menon V. Uddin L. Q. (2010). Saliency, switching, attention and control: a network model of insula function. *Brain Struct. Funct.* 214 655–667. 10.1007/s00429-010-0262-0 20512370 PMC2899886

[B85] Miu A. C. Piţur S. Szentágotai-Tătar A. (2016). Aesthetic emotions across arts: a comparison between painting and music. *Front. Psychol.* 6:1951. 10.3389/fpsyg.2015.01951 26779072 PMC4700299

[B86] Moore E. O. (1981). A prison environment’s effect on health care service demands. *J. Environ. Syst.* 11 17–34. 10.2190/KM50-WH2K-K2D1-DM69 22612255

[B87] Moran J. M. Kelley W. M. Heatherton T. F. (2013). What can the organization of the brain’s default mode network tell us about self-knowledge? *Front. Hum. Neurosci.* 7:391. 10.3389/fnhum.2013.00391 23882210 PMC3713343

[B88] Nadal M. Chatterjee A. (2019). Neuroaesthetics and art’s diversity and universality. *Wiley Int. Rev. Cogn. Sci.* 10:e1487. 10.1002/wcs.1487 30485700

[B89] Nodine C. Mello-Thoms C. Krupinski E. Locher P. (2008). Visual interest in pictorial art during an aesthetic experience. *Spat. Vis.* 21 55–77. 10.1163/156856807782753868 18073051

[B90] Panksepp J. Biven L. (2012). “A meditation on the affective neuroscientific view of human and animalian Mind Brains,” in *From the couch to the lab: trends in psychodynamic neuroscience*, eds Fotopoulou A. Pfaff D. Conway M. A., 145–175. 10.1093/med/9780199600526.003.0009

[B91] Pavlović M. Marković S. (2012). Automatic processes in aesthetic judgment: insights from the implicit association test. *Psihologija* 45 377–393. 10.2298/PSI1204377P

[B92] Pinho A. L. Ullén F. Castelo-Branco M. Fransson P. de Manzano Ö (2015). Addressing a paradox: dual strategies for creative performance in introspective and exterospective networks. *Cerebral Cortex* 26 3052–3063. 10.1093/cercor/bhv130 26088973

[B93] Plato (1995). *Phaedrus*, trans. Nehamas A. Woodruff P. (Indianapolis, IN: Hackett).

[B94] Prinz J. (2004). Can critics be dispassionate? The role of emotion in aesthetic judgment. In a Meeting of the American Society for Aesthetics, Houston, Texas.

[B95] Raichle M. E. (2015). The brain’s default mode network. *Ann. Rev. Neurosci.* 38 433–447. 10.1146/annurev-neuro-071013-014030 25938726

[B96] Raichle M. E. MacLeod A. M. Snyder A. Z. Powers W. J. Gusnard D. A. Shulman G. L. (2001). A default mode of brain function. *Proc. Natl. Acad. Sci.* 98 676–682. 10.1073/pnas.98.2.676 11209064 PMC14647

[B97] Ramachandran V. S. Hirstein W. (1999). The science of art: a neurological theory of aesthetic experience. *J. Conscious. Stud.* 6 15–51.

[B98] Reber R. Schwarz N. Winkielman P. (2004). Processing fluency and aesthetic pleasure: is beauty in the perceiver’s processing experience? *Personality Soc. Psychol. Rev.* 8 364–382. 10.1207/s15327957pspr0804_3 15582859

[B99] Rhodes G. (2006). The evolutionary psychology of facial beauty. *Annu Rev Psychol*. 57, 199–226. 10.1146/annurev.psych.57.102904.190208 16318594

[B100] Richards W. Jepson A. Feldman J. (1996). “Priors, preferences and categorical percepts,” in *Perception as bayesian inference*, eds. Knill D. Richards W. (Cambridge University Press), 93–122. 10.1017/CBO9780511984037.005

[B101] Roeske T. Larrouy-Maestri P. Sakamoto Y. Poeppel D. (2020). Listening to birdsong reveals basic features of rate perception and aesthetic judgements. *Proc. R. Soc. B* 287:3010. 10.1098/rspb.2019.3010 32208834 PMC7126030

[B102] Rudd M. Vohs K. D. Aaker J. (2012). Awe expands people’s perception of time, alters decision making, and enhances well-being. *Psychol. Sci.* 23 1130–1136. 10.1177/0956797612438731 22886132

[B103] Ryan R. M. Di Domenico S. I. (2016). “Distinct motivations and their differentiated mechanisms: reflections on the emerging neuroscience of human motivation,” in *Advances in motivation and achievement: recent developments in neuroscience research on human motivation*, eds Kim S. Reeve J. Bong M. (Emerald Group Publishing), 349–369. 10.1108/S0749-742320160000019009

[B104] Sarasso P. Neppi-Modona M. Sacco K. Ronga I. (2020). “Stopping for knowledge”: the sense of beauty in the perception-action cycle. *Neurosci. Biobehav. Rev.* 118 723–773. 10.1016/j.neubiorev.2020.09.004 32926914

[B105] Scarry E. (1999). *On beauty and being just.* Princeton, NJ: Princeton University Press. 10.1515/9781400847358

[B106] Scarry E. (2001). *Dreaming by the book.* Princeton, NJ: Princeton University Press.

[B107] Sharinger M. Wagner V. Knoop C. A. Menninghaus W. (2022). Melody in poems and songs: fundamental statistical properties predict aesthetic evaluation. *Psychol. Aesthe. Creat. Arts* 2022:465. 10.1037/aca0000465

[B108] Schmidhuber J. (2010). Formal theory of creativity, fun, and intrinsic motivation (1990–2010). *IEEE Trans. Autono. Mental Dev.* 2 230–247. 10.1109/TAMD.2010.2056368

[B109] Schoeller F. (2015). Knowledge, curiosity, and aesthetic chills. *Front. Psychol.* 6:1546. 10.3389/fpsyg.2015.01546 26539133 PMC4611094

[B110] Scrima F. Foddai E. Hamel J.-F. Carrein-Lerouge C. Codou O. Montalan B. (2022). Workplace aesthetic appreciation and exhaustion in a COVID-19 vaccination center: the role of positive affects and interest in art. *Int. J. Environ. Res. Public Health* 19:14288. 10.3390/ijerph192114288 36361164 PMC9654670

[B111] Seeley W. W. Menon V. Schatzberg A. F. Keller J. Glover G. H. Kenna H. (2007). Dissociable intrinsic connectivity networks for salience processing and executive control. *J. Neurosci.* 27 2349–2356. 10.1523/JNEUROSCI.5587-06.2007 17329432 PMC2680293

[B112] Shaftesbury A. C. (1999). *Characteristics of men, manners, opinions, times.* Cambridge: Cambridge University Press. 10.1017/CBO9780511803284

[B113] Silva P. J. Barona C. M. (2009). Do people prefer curved objects? Angularity, expertise, and aesthetic preference. *Emp. Stud. Arts* 27 25–42. 10.2190/EM.27.1.b 28491269

[B114] Simonyan K. Zisserman A. (2015). Very deep convolutional networks for large-scale image recognition. *arXiv* [preprint].

[B115] Smith B. H. (1968). *Poetic closure: a study of how poems end.* Chicago: University of Chicago Press.

[B116] Sparkman R. Austin L. M. (1980). The effect on sales of color in newspaper advertisements. *J. Advert.* 9 39–42. 10.1080/00913367.1980.10673336 1411820

[B117] Sporns O. Tononi G. Kötter R. (2005). The human connectome: a structural description of the human brain. *PLoS Comput. Biol.* 1:e42. 10.1371/journal.pcbi.0010042 16201007 PMC1239902

[B118] Spreng R. N. Mar R. A. Kim A. S. (2009). The common neural basis of autobiographical memory, prospection, navigation, theory of mind, and the default mode: a quantitative meta-analysis. *J. Cogn. Neurosci.* 21 489–510. 10.1162/jocn.2008.21029 18510452

[B119] Spreng R. N. Sepulcre J. Turner G. R. Stevens W. D. Schacter D. L. (2013). Intrinsic architecture underlying the relations among the default, dorsal attention, and frontoparietal control networks of the human brain. *J. Cogn. Neurosci.* 25 74–86. 10.1162/jocn_a_00281 22905821 PMC3816715

[B120] Sridharan D. Levitin D. J. Menon V. (2008). A critical role for the right fronto-insular cortex in switching between central-executive and default-mode networks. *Proc. Natl. Acad. Sci. U.S.A.* 105 12569–12574. 10.1073/pnas.0800005105 18723676 PMC2527952

[B121] Starr G. G. (2015). “Theorizing imagery, aesthetics, and doubly directed states,” in *The Oxford handbook of cognitive literary studies*, ed. Zunshine L. (Oxford University Press), 246–268.

[B122] Starr G. G. (2023a). “Goe and catch a falling star”: embodiment, cognition, and imagery,” in *The Routledge companion to literature and art*, eds Wang M. Murphy N. Lee C. J. (Routledge).

[B123] Starr G. G. (2023b). *Just in time: Temporality, aesthetic experience, and cognitive neuroscience.* Cambridge, MA: MIT Press.

[B124] Starr G. G. Belfi A. M. (2020). “Pleasure” in *Further reading*, eds Rubery M. Price L. (Oxford University Press), 282–293. 10.1093/oxfordhb/9780198809791.013.24

[B125] Stolnitz J. (1978). “The aesthetic attitude” in the rise of modern aesthetics. *J. Aesthe. Art Crit.* 36 409–422. 10.2307/430481

[B126] Takano R. Nomura M. (2022). Neural representations of awe: distinguishing common and distinct neural mechanisms. *Emotion* 22 669–677. 10.1037/emo0000771 32496077

[B127] Tartt D. (2016). *The goldfinch: a novel.* Boston,MA: Little, Brown.

[B128] Tsur R. (2012). *Poetic rhythm: structure and performance: an empirical study in cognitive poetics.* Eastbourne: Sussex Academic Press. 10.2307/j.ctv30c9fct

[B129] Ulrich R. S. (1979). Visual landscapes and psychological well-being. *Land. Res.* 4 17–23. 10.1080/01426397908705892

[B130] Ulrich R. S. (1984). View through a window may influence recovery from surgery. *Science* 224, 420–421. 10.1126/science.6143402 6143402

[B131] Ulrich M. Keller J. Hoenig K. Waller C. Grön G. (2014). Neural correlates of experimentally induced flow experiences. *Neuroimage* 86, 194–202. 10.1016/j.neuroimage.2013.08.019 23959200

[B132] Ulrich R. S. Zimring C. Zhu X. DuBose J. Seo H.-B. Choi Y.-S. (2008). A review of the research literature on evidence-based design. *HERD* 1, 61–125. 10.1177/193758670800100306 21161908

[B133] Van de Cruys S. Wagmans J. (2011). Putting reward in art: a tentative prediction error account of visual art. *Perception* 2 1035–1062. 10.1068/i0466aap 23145260 PMC3485793

[B134] Van de Cruys S. Brevoets J. Moors A. (2022). “Preferences need inferences learning, valuation, and curiosity in aesthetic experience,” in *The Routledge international handbook of neuroaesthetics*, eds. Skov M. Nadal M. (Routledge), 475–506. 10.4324/9781003008675-28

[B135] Van den Heuvel M. P. Sporns O. (2011). Rich-club organization of the human connectome. *J. Neurosci.* 31 15775–15786. 10.1523/JNEUROSCI.3539-11.2011 22049421 PMC6623027

[B136] Vartanian O. (2019). “Neuroscience of creativity,” in *The Cambridge handbook of creativity*, eds. Kaufman J. C. Sternberg R. J. (Cambridge University Press), 148–172. 10.1017/9781316979839.010

[B137] Vartanian O. Beatty E. L. Smith I. Blackler K. Lam Q. Forbes S. (2018). One-way traffic: the inferior frontal gyrus controls brain activation in the middle temporal gyrus and inferior parietal lobule during divergent thinking. *Neuropsychologia* 118 68–78. 10.1016/j.neuropsychologia.2018.02.024 29477840

[B138] Vessel E. A. Maurer N. Denker A. H. Starr G. G. (2018). Stronger shared taste for natural aesthetic domains than for artifacts of human culture. *Cognition* 179 121–131. 10.1016/j.cognition.2018.06.009 29936343

[B139] Vessel E. A. Starr G. G. Rubin N. (2012). The brain on art: intense aesthetic experience activates the default mode network. *Front. Hum. Neurosci.* 6:66. 10.3389/fnhum.2012.00066 22529785 PMC3330757

[B140] Vessel E. A. Starr G. G. Rubin N. (2013). Art reaches within: aesthetic experience, the self and the default mode network. *Front. Neurosci.* 7:258. 10.3389/fnins.2013.00258 24415994 PMC3874727

[B141] Walton K. L. (1990). *Mimesis as make-believe: on the foundations of the representational arts.* Cambridge, MA: Harvard University Press. 10.2307/2108134

[B142] Welke D. Purton I. Vessel E. A. (2021). Inspired by art: higher aesthetic appeal elicits increased felt inspiration in a creative writing task. *Psychol. Aesthe. Creat. Arts* 2021:393. 10.1037/aca0000393

[B143] Wilkins R. W. Hodges D. A. Laurienti P. J. Steen M. Burdette J. H. (2014). Network science and the effects of music preference on functional brain connectivity: from Beethoven to Eminem. *Sci. Rep.* 4 61–68. 10.1038/srep06130 25167363 PMC5385828

[B144] Williams P. G. Johnson K. T. Curtis B. J. King J. B. Anderson J. S. (2018). Individual differences in aesthetic engagement are reflected in resting-state fMRI connectivity: Implications for stress resilience. *NeuroImage* 179 156–165. 10.1016/j.neuroimage.2018.06.042 29908310 PMC6410354

[B145] Witzel C. Valkova H. Hansen T. Gegenfurtner K. R. (2011). Object knowledge modulates colour appearance. *Perception* 2 13–49. 10.1068/i0396 23145224 PMC3485772

[B146] Wundt W. (1874). “Grundzüge der physiologischen psychologie,” in *Emotions evoked by the sound of music: characterization, classification, and measurement*, eds Zentner M. Grandjean D. Scherer K. R.. 10.1037/1528-3542.8.4.494 18729581

[B147] Zentner M. Grandjean D. Scherer K. R. (2008). Emotions evoked by the sound of music: characterization, classification, and measurement. *Emotion* 8:494. 10.1037/1528-3542.8.4.49 18729581

[B148] Zhang J. W. Howell R. T. Iyer R. (2014). Engagement with natural beauty moderates the positive relation between connectedness with nature and psychological well-being. *J. Environ. Psychol.* 38 55–63. 10.1016/j.jenvp.2013.12.013

