# Corrigendum: Aesthetic experience models human learning

**DOI:** 10.3389/fnhum.2024.1356631

**Published:** 2024-02-13

**Authors:** G. Gabrielle Starr

**Affiliations:** Department of Neuroscience and English, Pomona College, Claremont, CA, United States

**Keywords:** neuroaesthetics, learning, aesthetics, pleasure, preference, default mode network, salience network, central executive network

In the published article there was an error in Section 4, **Aesthetics, expectation, and Bayesian prediction**, paragraph three, which misstated a component of the findings of Cheung et al. (2019). The sentence stated: “The second key is that Cheung et al. (2019) found that individuals experienced high pleasure when an unexpected chord came in the context of high uncertainty: the potential to gain information—to learn—is here at its highest because baseline predictability is so low (there is not enough knowledge to enable a confident prediction).”

This should have been written as:

“The second key is that Cheung et al. (2019) found that individuals experienced high pleasure when an unexpected chord came in the context of high certainty: the potential to gain information—to learn—is significant because baseline predictability was high (listeners can update a previously confident prediction).”

The author apologizes for this error and state that this does not change the scientific conclusions of the article in any way. The original article has been updated.

## References

[B1] Cheung V. K. Harrison P. M. Meyer L. Pearce M. T. Haynes J. D. Koelsch S. (2019). Uncertainty and surprise jointly predict musical pleasure and amygdala, hippocampus, and auditory cortex activity. Curr. Biol. 29, 4084–4092. 10.1016/j.cub.2019.09.067 31708393

